# Rebound of relapses after discontinuation of rituximab in a patient with MOG-IgG1 positive highly relapsing optic neuritis: a case report

**DOI:** 10.1186/s12883-018-1222-1

**Published:** 2018-12-21

**Authors:** Seok-Jin Choi, Boram Kim, Haeng-Jin Lee, Seong-Joon Kim, Sung-Min Kim, Jung-Joon Sung

**Affiliations:** 1 Department of Neurology, Inha University Hospital, Incheon, Republic of Korea; 2 Department of Neurology, Seoul National University Hospital, 101, Daehak-Ro Jongno-Gu, Seoul, 03080 Republic of Korea; 3 Department of Ophthalmology, Seoul National University Hospital, Seoul, Republic of Korea

**Keywords:** MOG-IgG1, Optic neuritis, Highly relapsing, Rituximab

## Abstract

**Background:**

Myelin oligodendrocyte glycoprotein immunoglobulin G1 (MOG-IgG1)-associated disease is suggested as a separate disease entity distinct from multiple sclerosis and neuromyelitis optica spectrum disorder. Nonetheless, the optimal treatment regimen for preventing relapses in MOG-IgG1-associated disease remains unclear.

**Case presentation:**

We describe the case of a 45-year-old man with MOG-IgG1-positive highly relapsing optic neuritis who had experienced 5 attacks over 21 months and had monocular blindness despite prednisolone and azathioprine therapy. He began treatment with rituximab, which reduced the rate of relapse markedly. Following discontinuation of rituximab, however, the patient experienced two successive optic neuritis attacks 2 and 4 months after B-lymphocyte restoration.

**Conclusions:**

Highly relapsing MOG-IgG1-associated disease can be prevented with rituximab even when the MOG-IgG1 titers are relatively stationary. Discontinuation of rituximab and restoration of B-lymphocytes may be associated with the rebound of disease activity.

## Background

Myelin oligodendrocyte glycoprotein immunoglobulin G1 (MOG-IgG1)-associated disease is suggested as a separate disease entity distinct from multiple sclerosis and neuromyelitis optica spectrum disorder (NMOSD) with anti-aquaporin-4 IgG (AQP4-IgG); it has a predilection for the optic nerve rather than spinal cord, perineural enhancement extending to adjacent soft tissues on magnetic resonance imaging (MRI), and a less unfavorable prognosis than NMOSD [1]. Recent studies with a sufficient number of patients and duration of follow-up have indicated that a considerable number of patients with MOG-IgG1 have relapsing attacks in the central nervous system followed by neurological deficits [2, 3]. Nonetheless, the optimal treatment regimen for preventing relapses in patients with MOG-IgG1-associated disease has only recently begun to be studied [4]. Here, we describe a patient with highly relapsing optic neuritis (ON) associated with MOG-IgG1, whose ON attacks were relatively well-prevented with rituximab (RTX) treatment. However, the patient experienced rebounds of repeated ON attacks shortly after the restoration of B-cells following discontinuation of RTX.

## Case presentation

**Fig. 1:**
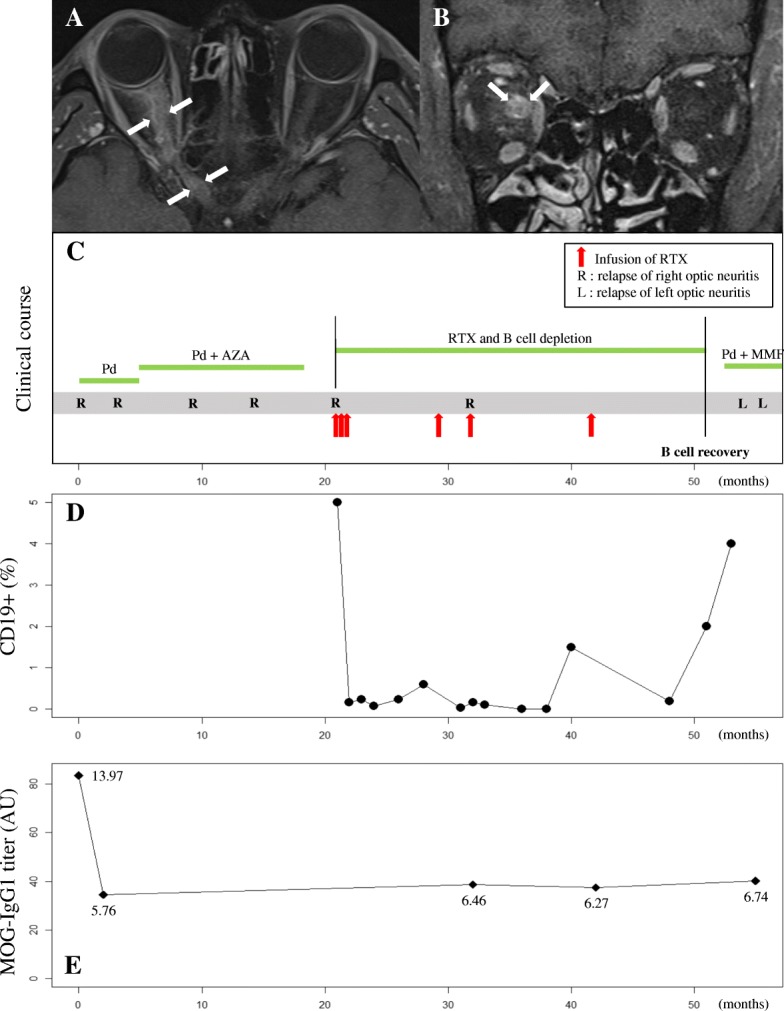
(**a**) Axial and (**b**) coronal T1-weighted magnetic resonance images demonstrating diffuse gadolinium enhancement and swelling along the right anterior and posterior optic nerve. **c** Longitudinal clinical course of recurrent optic neuritis. **d** Change of CD19+ B-lymphocytes (%) during rituximab treatment. **e** Change of MOG-IgG1 titers measured by a geometric mean fluorescence (G-mean) ratio of the MOG-expressing cells that bound to IgG1 using in-house flow cytometry (G-mean ratio = G-mean values of the patient’s sera / G-mean values of the healthy control)

A 45-year-old man presented with decreased right visual acuity (VA) accompanied by periocular pain lasting for 1 week. Ophthalmological examination revealed that the patient’s right eye was only able to perceive light (best-corrected VA, light perception/0.9 in decimals, measured using a Snellen chart) and had relative afferent pupillary defect of grade 3, diffuse disc swelling, and inferior disc hemorrhage. Neurological examination showed normal muscle strength in all extremities, no sensory deficits, normoactive deep tendon reflexes, and no signs of bladder or bowel dysfunction. Orbit MRI revealed T2 high signal intensities and diffuse contrast enhancement along the right anterior and posterior optic nerve, as well as perineural enhancement [1] (Fig. 1-a and b). The results of cerebrospinal fluid (CSF) analysis showed a red blood cell count of 0/μL, a white blood cell count of 1/μL, and a protein level of 27 mg/dL. CSF oligoclonal band measured by isoelectric focusing was negative and IgG index was 0.64. The result of a serum AQP4-IgG flow cytometry assay using AQP4-M23-expressing live cells was negative [5]. Right ON was suspected, and intravenous methylprednisolone (1000 mg pulse therapy) for 5 days followed by oral prednisolone (60 mg daily) were prescribed. The right VA of the patient was improved to 0.5 (visual Functional System score improved to 2 from 5).

**Fig. 2:**
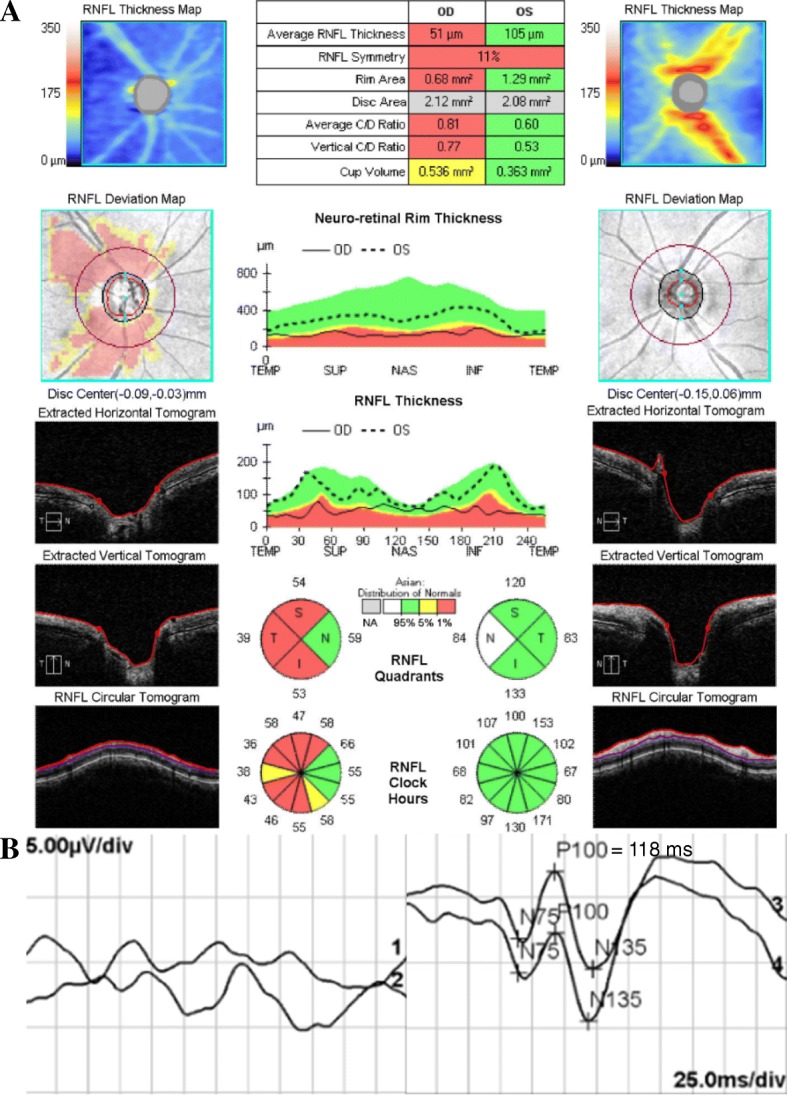
**a** (Remission state after the second optic neuritis) the average retinal nerve fiber layer thickness measured by spectral-domain optical coherence tomography was decreased in the right eye (right 51 μm and left 105 μm), with preferential thinning of the superior, temporal, and inferior quadrants. **b** (During fifth optic neuritis) the pattern-reversal visual evoked potential showed an abnormal waveform in the right eye with diminished amplitude. The left eye presented a relatively preserved response with prolonged P100 latency (118 ms)

The second right ON attack (0.15/1.0) occurred 4 months after the first ON when the prednisolone dose had been tapered to 10 mg daily. Thus, azathioprine 50 mg twice per day was started in a remission state between the second and third ON (4 months prior to the third ON). The average thickness of a retinal nerve fiber layer measured by spectral-domain optical coherence tomography was decreased in the right eye (right 51 μm and left 105 μm) (Fig. 2-a). The third (hand movement/0.9) and fourth (finger count/1.2) right ON attacks occurred 6 and 10 months after the second ON, respectively, while the prednisolone dose was maintained at 5 mg daily and azathioprine was 75 mg twice per day. Following these attacks, the patient developed left central serous chorioretinopathy (0.15/0.9) associated with long-term steroid use. The 25 mg dose of prednisolone was thus tapered out at this point. Nevertheless, right ON recurred 2 months later for a fifth time (hand movement/0.9) when the patient was under azathioprine treatment only. At this time, the patient developed monocular blindness. The pattern-reversal visual evoked potential showed an abnormal waveform in the right eye with diminished amplitude. The left eye presented a relatively preserved response with prolonged P100 latency (118 ms) (Fig. 2-b). Serum from the patient sampled at the time of the fifth ON attack was tested for MOG-IgG1 using a cell-based assay utilizing full-length human MOG (Radcliffe Hospital, Oxford, UK) [6]. The result of this test was positive.

Despite continued immunosuppressive treatment and due to the repeated ON attacks and the side effect of the steroid (chorioretinopathy), the patient was administered RTX (375 mg/m^2^, 3 weekly infusion for induction and 3 maintenance doses under CD19+ B-cell monitoring over 29 months). Although one mild ON attack (no light perception/1.2) occurred in the patient’s right eye during RTX treatment, the rate of relapse decreased markedly and the patient’s visual function was well-maintained. However, 32 months after the initiation of RTX treatment, we became unable to maintain RTX treatment due to insurance issues (denial for reimbursement). As a result, the treatment was switched to mycophenolate mofetil (250 mg twice per day) combined with oral prednisolone (5 mg every other day). The patient’s CD19+ B-lymphocyte level was restored to 2 and 4% at 9 and 11 months after the last RTX infusion, respectively. Subsequently, 2 more left ON attacks (hand movement/1.0 and hand movement/0.15) occurred within a one-month interval (Fig. 1-c and 1-D). The titer of MOG-IgG1 was measured by a geometric mean fluorescence (G-mean) ratio of the MOG-expressing cells bound to IgG1 using in-house flow cytometry. The G-mean ratio was calculated for each sera as followings: G-mean values of the patient’s sera / G-mean values of the healthy control. The titer was not associated with the continuation or cessation of the RTX treatment (Fig. 1-e).

## Discussion and conclusions

Here, we describe the longitudinal clinical course and treatment response to RTX therapy in a patient with MOG-IgG1-positive highly relapsing ON. We found that 1) highly relapsing MOG-IgG1-associated disease can be prevented with RTX even when the MOG-IgG1 titers are relatively preserved, and 2) discontinuation of RTX in patients with this condition can cause rebound of disease activity with restoration of B-lymphocytes.

Initial reports regarding MOG-IgG1-associated disease indicated that it typically has a monophasic and benign disease course [7]. However, recent multicenter studies have shown that a considerable proportion of patients have a relapsing course of disease, and some have significant neurological deficits [2, 3]. More recently, RTX was reported to reduce the rate of relapse in some cases of MOG-IgG1-associated disease [4]. Nevertheless, the results of studies comparing the patient’s condition before vs. after the treatment should be interpreted with caution because the disease may have a naturally decreasing relapse rate in the later stages, as in NMOSD [8], and also the statistical phenomenon of regression towards the mean. In this regard, the present case, wherein we observed a restoration of B-lymphocytes and a subsequent rebound of relapses after discontinuation of RTX treatment, implies that long-term RTX maintenance therapy may be helpful in patients with highly relapsing MOG-IgG1-associated disease.

Despite initial treatment with azathioprine and prednisolone, the patient had a high relapse rate of 0.238/year (5 attacks over 21 months) and subsequent unilateral visual loss in the right eye. After initiating RTX treatment, his relapse rate markedly decreased to 0.031/year (1 attack over 32 months). However, the patient experienced 2 ON attacks over 4 months following cessation of RTX treatment and restoration of B-lymphocytes.

In summary, the case described here illustrates that RTX can be a good treatment option for preventing relapses in MOG-IgG1-associated disease. The treatment effect was observed despite the relatively unchanged MOG-IgG1 titers during the treatment period. Finally, cessation of RTX treatment and restoration of B-lymphocytes may be associated with the rebound of disease activity. RTX may serve as an effective treatment regimen in MOG-IgG1-associated disease, especially in patients with high relapse rates.

## Abbreviations

AQP4: anti-aquaporin-4
CSF: cerebrospinal fluid
IgG: immunoglobulin G
MOG: myelin oligodendrocyte glycoprotein
MRI: magnetic resonance imaging
NMOSD: neuromyelitis optica spectrum disorder
ON: optic neuritis
RTX: rituximab
VA: visual acuity
